# The Forms of the Lectin Tff2 Differ in the Murine Stomach and Pancreas: Indications for Different Molecular Functions

**DOI:** 10.3390/ijms24087059

**Published:** 2023-04-11

**Authors:** Eva B. Znalesniak, Aikaterini Laskou, Franz Salm, Katharina Haupenthal, Sönke Harder, Hartmut Schlüter, Werner Hoffmann

**Affiliations:** 1 Institute of Molecular Biology and Medicinal Chemistry, Otto-von-Guericke University Magdeburg, Leipziger Str. 44, 39120 Magdeburg, Germany; 2 Section Mass Spectrometry and Proteomics, Diagnostic Center, University Medical Center Hamburg-Eppendorf, Martinistr. 52, 20246 Hamburg, Germany

**Keywords:** trefoil factor, TFF2, lectin, stomach, pancreas, mucin, MUC6, pancreatic cancer, branching morphogenesis, pancreatic organogenesis

## Abstract

The lectin TFF2 belongs to the trefoil factor family (TFF). This polypeptide is typically co-secreted with the mucin MUC6 from gastric mucous neck cells, antral gland cells, and duodenal Brunner glands. Here, TFF2 fulfills a protective function by forming a high-molecular-mass complex with the MUC6, physically stabilizing the mucus barrier. In pigs and mice, and slightly in humans, TFF2 is also synthesized in the pancreas. Here, we investigated the murine stomach, pancreas, and duodenum by fast protein liquid chromatography (FPLC) and proteomics and identified different forms of Tff2. In both the stomach and duodenum, the predominant form is a high-molecular-mass complex with Muc6, whereas, in the pancreas, only low-molecular-mass monomeric Tff2 was detectable. We also investigated the expression of *Tff2* and other selected genes in the stomach, pancreas, and the proximal, medial, and distal duodenum (RT-PCR analysis). The absence of the Tff2/Muc6 complex in the pancreas is due to a lack of Muc6. Based on its known motogenic, anti-apoptotic, and anti-inflammatory effects, we propose a protective receptor-mediated function of monomeric Tff2 for the pancreatic ductal epithelium. This view is supported by a report that a loss of *Tff2* promotes the formation of pancreatic intraductal mucinous neoplasms.

## 1. Introduction

TFF2 (formerly: (pancreatic) spasmolytic polypeptide [1,2]) is a secretory polypeptide belonging to the trefoil factor family (TFF; reviews: [3,4]). Its exocrine secretion predominantly occurs in gastric mucous neck cells, antral gland cells, and duodenal Brunner glands cells, together with the mucin MUC6 [5,6,7,8]. In the mouse, and not in the human, Tff2 is the predominant TFF peptide in the lung [9,10]. In some species (such as pig and mouse), it is clearly expressed in the pancreas [2,11,12,13,14], exclusively in the exocrine acini of the glands, as shown for the pig [15] and the mouse [14]. From here, TFF2 reaches the duodenum via the pancreatic duct and the ampulla of Vater as a constituent of the pancreatic juice. As known from the porcine pancreas, TFF2 secretion is highly regulated and very sensitive to physiological stimuli [16]. Its release into the pancreatic juice is controlled by parasympathetic mechanisms, which also stimulate in parallel the secretion of a cocktail of pancreatic food-digesting enzymes [16]. One of the major physiological stimuli is the ingestion of food [16].

In humans, TFF2 was reported as not being detectable in a normal pancreas [17,18] or in the ampulla of Vater (also known as hepatopancreatic ampulla) [7]. However, small amounts of TFF2 are probably restricted to the human exocrine pancreatic ducts [19,20], and minute amounts of TFF2 and MUC6 are secreted from the pancreatic duct glands (PDGs) of the human and mouse, mainly found in the proximal and central pancreatic duct epithelium [21]. Of special note, these PDGs harbor progenitor cells responsible for pancreatic ductal epithelial repair [21].

In addition to its exocrine secretion, there is also endocrine secretion of TFF2 (review: [4]), in particular in lymphoid tissues (spleen, lymph nodes, and thymus [22,23]) and the CNS [24]. Furthermore, aberrant and partially ectopic expression of TFF peptides is observed during pathological conditions, such as inflammation, a specific metaplasia (spasmolytic polypeptide expressing metaplasia, SPEM), and tumors (reviews: [6,25,26]). For example, TFF2 is upregulated in a murine pancreatitis model and in human pancreatitis, [20,21] as well as in pancreatic carcinoma [27].

**Figure 1:**
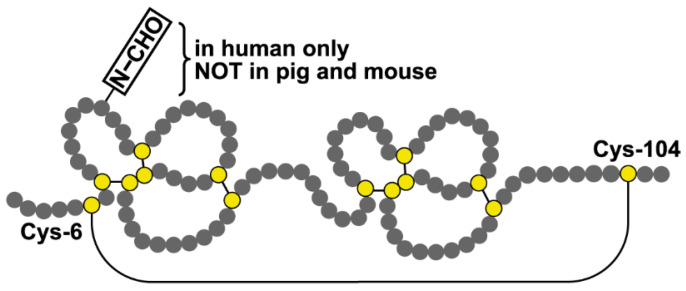
Disulfide-linked schematic structure of TFF2. Cysteine residues are shown in yellow. The N-linked carbohydrate moiety typical of human TFF2 is indicated.

Mammalian TFF2 contains two cysteine-rich TFF domains, and the N- and C-terminal regions are connected with an additional disulfide bridge between Cys-6 and Cys-104, generating a circular structure (Figure 1; reviews: [2,28]). Human TFF2 is N-glycosylated, containing in its gastric form an unusual fucosylated *N*,*N’*-diacetyllactosediamine (LacdiNAc) oligosaccharide [29], whereas the porcine and murine homologous polypeptides are not N-glycosylated (Figure 1). The extent of N-glycosylation, as well as the concentration of TFF2 in the human gastric juice, shows dramatic diurnal variations [30]. There is also an ortholog of TFF2, termed xP4, in the frog *Xenopus laevis*, which contains four TFF domains arranged in tandem [31,32]. Furthermore, a peptide with astonishing structural similarity to TFF2 exists in *X. laevis* skin, also consisting of two TFF domains [33].

The molecular function of TFF2 in the stomach (and the duodenum) has been established as a lectin, which binds specifically to the terminal carbohydrate moiety of the mucin MUC6, i.e., the GlcNAcα1→4Galβ1→R epitope (review: [34]). This glycotope is evolutionarily conserved in MUC6 from frog to human [35], which explains why porcine TFF2 even binds to the *X. laevis* ortholog of MUC6 [36]. The key enzyme for the synthesis of the unusual terminal αGlcNAc residue is α1,4-*N*-acetylglucosaminyltransferase (α4GnT; encoded by the *A4gnt* gene in the mouse), which shows the same highly restricted expression pattern as MUC6 and TFF2, i.e., gastric mucous neck cells, antral gland cells, and duodenal Brunner glands (review: [37]). The αGlcNAc residue is recognized by the lectin GSA-II from *Griffonia simplicifolia* [35]. Thus, GSA-II is often used for histochemical staining of MUC6 in both gastric and Brunner glands [38,39].

The physical interaction of TFF2 and MUC6 probably creates a crosslinked mucous network, which affects the viscoelastic properties of mucous gels, as shown in vitro [40], and even prevents shrinking of secretory granules during the processing of the samples for electron microscopy [41]. This explains why, in the alternating mucin layers of the gastric mucus, TFF2 is strictly co-localized with MUC6 [8,42]. Taken together, by crosslinking MUC6, TFF2 is an integral structural constituent of the gastroduodenal mucus barrier, the latter physically protecting the cells from acid-induced damage and bacterial infection [4,28,43,44].

In addition to being a structural component of the gastroduodenal mucus, various biological activities have been described for monomeric TFF2 in vitro, indicating a receptor-mediated function, such as enhancement of cell migration (motogenic effect) and inhibition of apoptosis (reviews: [3,4,6]). For example, TFF2 weakly enhanced the chemotaxis of bronchial BEAS-2B cells [45,46,47], as well as pancreatic cell lines [20], and inhibited apoptosis in several cancer cell lines [48,49] and pancreatic explant cultures [14]. Both of these effects are perfectly suited to synergistically enhance the rapid repair of epithelia by cell migration, a process termed “restitution” (reviews: [3,4,50]). Cell migration and survival mechanisms are even coordinately regulated [51]. Of note, these effects are observed at concentration ranges of 5 × 10^−8^–10^−6^ M or above only, excluding TFF2 as a classical high-affinity peptide ligand (review: [4]). Thus, rather low-affinity interactions typical of lectins were proposed to modulate a variety of glycosylated receptors (“lectin-triggered receptor activation/blocking hypothesis” [25,28,52]). A similar mechanism was described for galectins [53], and it would also explain the role of TFF2 as an anti-inflammatory factor and for the promotion of Th2 immunity (reviews: [22,25]). For example, TFF2 was reported, e.g., to block activation of the interleukin (IL)-1β receptor in macrophages [23], to antagonize the IL-12 release from macrophages (probably via a Toll-like receptor, TLR), or to induce IL-13 expression in gastric surface mucous cells (review: [25]). In addition to the IL-1β receptor and a TLR, CXCR4 might be one of the putative TFF2 receptors [54,55], together with a plethora of other transmembrane glycoproteins, such as β1-integrin, CRP-ductin/DMBT1^gp340^, proteinase-activated receptor PAR4, and LINGO3 (reviews: [3,4,25]).

There are also indications of the existence of different Tff2 receptors in embryonic pancreatic cells, with biological responses at a Tff2 concentration of 3 × 10^−7^ M [14]. Tff2 was detectable starting at E16.5 in developing acinar cells [14]. At that time, secondary transition branching morphogenesis was observed (review: [56]). A combination of diffusible protein ligands and specific receptors plays a key role in this process. Remarkably, Tff2 prevented the apoptosis of insulin-producing cells and Nkx6.1^+^ precursor cells through CXCR4 and a yet unidentified Tff2 receptor, respectively [14]. These results are reminiscent of those describing an anti-apoptotic effect, as well as branching morphogenesis (tubulogenesis), promoted by TFF2 (at concentrations of 5 × 10^−8^ M to 10^−7^ M) in a cancer cell line [48]. The effect of TFF2 on branching morphogenesis would be ideally suited to support murine pancreatic organogenesis, probably together with other factors [56]. Thus, a detailed analysis of the pancreas of *Tff2*^KO^ mice would be highly interesting as changes might also explain the protection of *Tff2*^KO^ mice from high-fat-induced obesity [57], e.g., due to the down-regulation of pancreatic lipases.

Tff2-deficient (*Tff2*^KO^) mice do not show a severe phenotype (reviews: [4,22,25]). They have increased susceptibility to indomethacin-induced ulcerations [58], *Helicobacter pylori*-induced gastritis [59], and *Yersinia enterocolitica* infection, and their recovery after dextran sodium sulfate (DSS)-induced colitis is delayed (reviews: [4,25]). Macrophages of *Tff2*^KO^ mice are hyper-responsive to IL-1β stimulation and have an increased level of IL-12/23p40, and altered expression of immune-regulating genes [22,23]. Of note, when transgenic *Pdx1-Cre;LSL-KRAS*^G12D^ (KC) mice were bred with *Tff2*^KO^ mice, the KC/*Tff2*^KO^ offspring developed a greater number and higher grade of pancreatic intraepithelial neoplasia than the KC mice, indicating a tumor-suppressor activity (protective effect) of Tff2 in the murine pancreas [60].

In the past, we discovered that the TFF2 forms in the porcine stomach and pancreas differ [61]. Gastric TFF2 was exclusively associated with the high-molecular-mass mucus fraction, whereas pancreatic TFF2 appeared as a low-molecular-mass homodimer [61]. Thus, it was the aim of the present study to systematically investigate the Tff2 forms in the murine stomach, pancreas, and duodenum, respectively, with the help of size-exclusion chromatography (SEC). We also performed expression profiling of selected genes (RT-PCR analysis) in order to explain why different Tff2 forms occur. We hypothesize that the different Tff2 forms are suggestive of different molecular functions, i.e., a possible receptor-mediated protective function of monomeric pancreatic Tff2 (e.g., motogenic, anti-apoptotic, anti-inflammatory) versus physical stabilization, particularly of the gastroduodenal mucus barrier (high-molecular-mass Tff2/Muc6 complex).

## 2. Results

### 2.1. Characterization of Tff2 Forms in the Murine Stomach, Pancreas, and Duodenum by SEC

**Figure 2:**
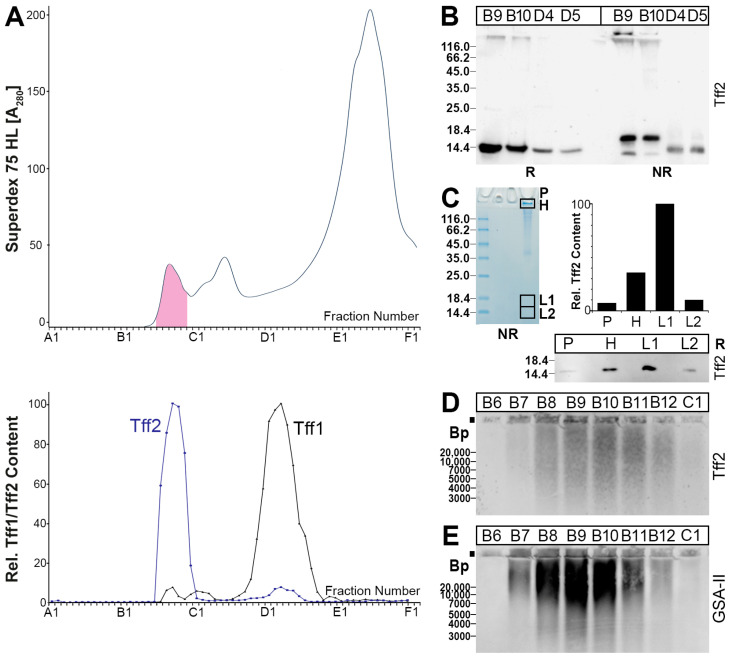
Analysis of a murine gastric extract (5 animals). (**A**) Elution profile after SEC (Superdex 75 HL) as determined by absorbance at 280 nm (PAS-positive mucin fractions: pink). Underneath: Distribution of the relative Tff1 (black) and Tff2 contents (blue) as determined by Western blot analysis under reducing conditions and semi-quantitative analysis of the monomeric band intensities. (**B**) 15% SDS-PAGE under reducing (R) and non-reducing (NR) conditions, respectively, and Western blot analysis of the high-molecular-mass fractions B9 and B10, as well as the low-molecular-mass fractions D4 and D5 concerning Tff2. (**C**) Non-reducing 15% SDS-PAGE of fraction B10 and Coomassie staining. The high- (H) and low-molecular-mass regions (L1, L2) were excised, proteins eluted, and subjected to reducing SDS-PAGE. The samples not entering the gel were removed from the gel pocket (P) and subjected to reducing SDS-PAGE. Western blot analysis concerning Tff2 of P, H, L1, and L2 and relative Tff2 content. (**D**,**E**) 1% AgGE and Western blot analysis of fractions B6–C1 concerning Tff2 (**D**) or Muc6 (lectin GSA-II, (**E**)). Relative standard: DNA ladder (base pairs).

A murine gastric extract was separated by SEC, and the Tff2 immunoreactivities were tested (Figure 2). The Tff2 content mainly peaked in the high-molecular-mass region (B9, B10), together with the periodic acid–Schiff (PAS)-positive mucins (Figure 2A). Only minor amounts were detectable in the low-molecular-mass region (peak: D2–D5). For comparison, the Tff1 content was also determined. The majority of Tff1 peaked in the low-molecular-mass region, and only a small amount of Tff1 was detectable in the mucin region (Figure 2A). After reducing SDS-PAGE, Tff2 appears as a monomer with a Mr of about 14k (Figure 2B). In contrast, after non-reducing SDS-PAGE, the Tff2 from B9 and B10 appears mainly as a double band, with a Mr of about 18k and a minor band with a Mr of about 14k; also, minor amounts of Tff2 were still visible in the high-molecular-mass region (Figure 2B). In contrast, the pattern of Tff2 from D4 and D5 under non-reducing conditions was different.

Porcine gastric TFF2 was previously shown to bind strongly to mucin, even after boiling in SDS (i.e., after non-reducing SDS-PAGE) [62]. To test whether murine gastric Tff2 also exists in high-molecular-mass forms after non-reducing SDS-PAGE, we analyzed the Tff2 content in the gel pocket, and the high- and low-molecular-mass regions (Figure 2C). The majority of Tff2 appeared in the 18k form (L1); however, Tff2 was also detectable in significant amounts in the high-molecular-mass region (H), and even in the gel pocket. After 1% agarose gel electrophoresis (AgGE), both Tff2 as well as the mucin Muc6 (lectin GSA-II) were detected in the PAS-positive high-molecular-mass fractions B7–B12 (Figure 2D,E). Here, the Tff2 and Muc6 appeared to be associated to form ultrahigh-molecular-mass complexes, which, in part, did not enter the agarose gel.

**Figure 3:**
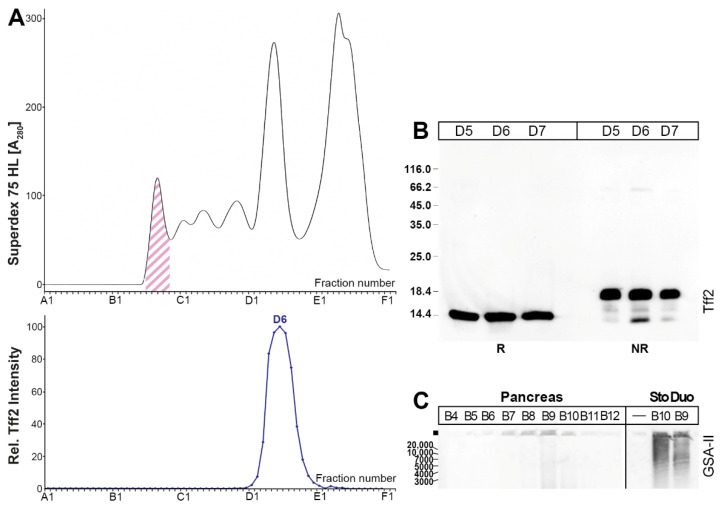
Analysis of a murine pancreatic extract (3 animals). (**A**) Elution profile after SEC on a Superdex 75 HL column as determined by absorbance at 280 nm (faint PAS-positive mucin fractions: pink-hashed). Underneath: Distribution of the relative Tff2 content as determined by Western blot analysis under reducing conditions and semi-quantitative analysis of the monomeric band intensities. (**B**) 15% SDS-PAGE under reducing (R) and non-reducing (NR) conditions, respectively, and Western blot analysis of the low-molecular-mass fractions D5–D7 concerning Tff2. (**C**) 1% AgGE and Western blot analysis of high-molecular-mass fractions B4–B12 concerning Muc6 (lectin GSA-II). As positive controls, equivalent samples from the stomach (Sto) and duodenum (Duo), respectively, were analyzed. Relative standard: DNA ladder (base pairs).

As a next step, a murine pancreatic extract was analyzed in a similar manner (Figure 3). Here, Tff2 was detectable in the low-molecular-mass region only (Figure 3A). After reducing SDS-PAGE, the Tff2 appears as a single band with a Mr of about 14k (Figure 3B), whereas, after non-reducing SDS-PAGE, the Tff2 appears as a triple band (major: 18k; minor 16k, 14k; Figure 3B). After AgGE, the Muc6 was not detectable in the high-molecular-mass range (Figure 3C).

**Figure 4:**
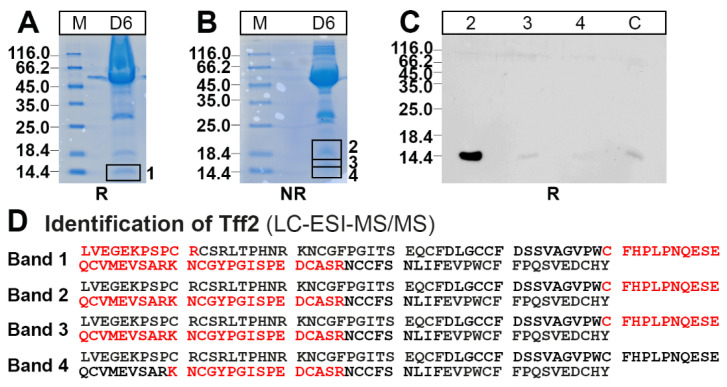
Proteome analysis of the low-molecular-mass form of Tff2 in a pancreatic extract (fraction D6 from Figure 3). (**A**) SDS-PAGE under reducing (R) conditions and Coomassie staining. Band 1 was excised. (**B**) NR SDS-PAGE and Coomassie staining. Bands 2, 3, and 4 were excised, proteins eluted, and subjected to reducing SDS-PAGE. (**C**) Western blot analysis concerning Tff2 (bands 2–4; C positive control). (**D**) Results of the proteome analyses after tryptic in-gel digestion of bands 1–4. Identified regions in Tff2 are shown in red.

In order to verify that the low-molecular-mass peak actually contains Tff2, fraction D6 (Figure 3) was separated by preparative reducing (Figure 4A) and non-reducing SDS- PAGE (Figure 4B), respectively. Bands 2–4 were eluted and tested for Tff2 by Western blotting under reducing conditions, indicating Tff2 predominantly in band 2 (Figure 4C). Furthermore, Tff2 was identified in all four bands by bottom-up proteomics (Figure 4D).

**Figure 5:**
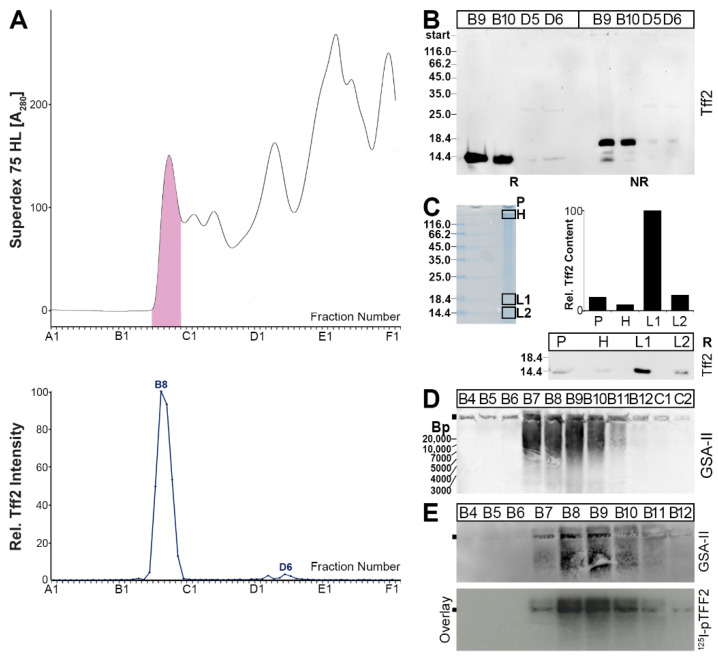
Analysis of a murine duodenal extract (complete duodena from 4 animals). (**A**) Elution profile after SEC on a Superdex 75 HL column as determined by absorbance at 280 nm (PAS-positive mucin fractions: pink). Underneath: Distribution of the relative Tff2 content as determined by Western blot analysis under reducing conditions and semi-quantitative analysis of the monomeric band intensities. (**B**) 15% SDS-PAGE under reducing (R) and non-reducing (NR) conditions, respectively, and Western blot analysis of the high-molecular-mass fractions B9 and B10, as well as the low-molecular-mass fractions D6 and D7 concerning Tff2. (**C**) Non-reducing 15% SDS-PAGE of fraction B8 and Coomassie staining. The high- (H) and low-molecular-mass regions (L1, L2) were cut out, the proteins were eluted, and subjected to reducing SDS-PAGE. Also, the remaining high-molecular-mass sample not entering the gel was removed from the gel pocket (P) and subjected to reducing SDS-PAGE. Western blot analysis concerning Tff2 of P, H, L1, and L2 and relative Tff2 content. (**D**) 1% AgGE and Western blot analysis of the fractions B4–C2 concerning Muc6 (lectin GSA-II). Relative standard: DNA ladder (base pairs). (**E**) 1% AgGE and Western blot analysis of high-molecular-mass fractions B3–B12 of a parallel SEC concerning Muc6 (lectin GSA-II). Shown are also the hybridization signals (autoradiography) obtained after incubating the blot with ^125^I-labeled porcine pancreatic TFF2 (pTFF2; overlay assay).

Finally, a duodenal extract (complete duodenum) was analyzed (Figure 5). Here, predominantly a high-molecular-mass form (peak: B8), congruent with the PAS-positive mucin range, was detected (Figure 5A). Furthermore, minute amounts of a low-molecular-mass form appeared (peak: D6; Figure 5A).

After the reducing SDS-PAGE, Tff2 from both the high- and low-molecular-mass range appeared as a 14k band (Figure 5B). After the non-reducing SDS-PAGE, two forms were detectable below 18k. To test whether duodenal Tff2 still exists in the high-molecular-mass forms after the non-reducing SDS-PAGE, we analyzed the Tff2 content in the gel pocket and the high- and low-molecular-mass regions (Figure 5C). The majority of the Tff2 appeared in the 18k form (L1); only minute amounts of Tff2 were detectable in the gel pocket (P) and the high-molecular-mass region (H) (Figure 5C). After AgGE, Muc6 was detectable (lectin GSA-II) in the PAS-positive high-molecular-mass region (fractions B7–B11; Figure 5D,E). Furthermore, ^125^I-labeled porcine pancreatic TFF2 (pTFF2) binds to the same regions (overlay assay; Figure 5E).

**Figure 6:**
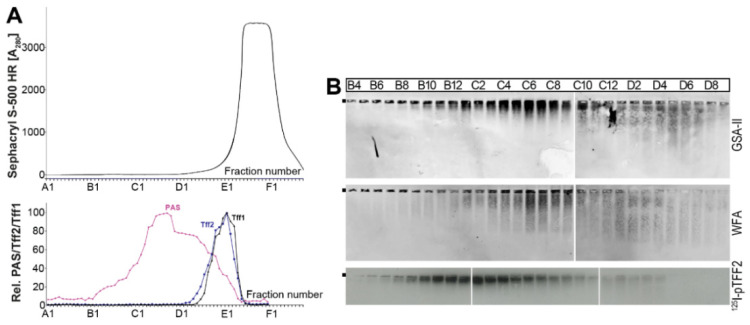
Analysis of a murine gastric extract (9 animals) after reduction in boiling β-mercaptoethanol. (**A**) Elution profile after SEC on a Sephacryl S-500 High Resolution column as determined by absorbance at 280 nm. Underneath: Distribution of the relative Tff1 (black) and Tff2 contents (blue). For comparison, the fractions were analyzed for their mucin content using the PAS reaction (pink). (**B**) 1% AgGE and subsequent Western blot analysis of the mucin-containing fractions B4–D9 concerning Muc6 (lectin GSA-II) and Muc5ac (lectin WFA), respectively. The hybridization signals (autoradiography) obtained after incubating the blot with ^125^I-labeled porcine pancreatic TFF2 (pTFF2; overlay assay) are also shown. The start is marked with a dot on the left.

In the past, human gastric TFF2 was shown to bind to the mucin MUC6 and not MUC5AC [43]. Similarly, as described previously for a human gastric extract [43], a murine gastric extract was reduced and denatured by boiling in 1% β-mercaptoethanol and separated by a HiPrep 16/60 Sephacryl S-500 High Resolution (S-500) column (Figure 6). The PAS-positive mucin peak was separated from the Tff2 (Figure 6A). According to a previous report [63], murine Muc6 was detected after 1% AgGE with the lectin GSA-II and murine Muc5ac with the lectin WFA from *Wisteria floribunda* (Figure 6B). The GSA-II and the WFA patterns were not congruent, indicating at least a partial separation of Muc6 and Muc5ac, similarly as described for the human mucins [43]. Furthermore, from the result of an overlay assay, it seems that ^125^I-pTFF2 predominantly binds to Muc6 (Figure 6B).

### 2.2. Expression Analysis of the Murine Stomach, Pancreas, and Duodenum (RT-PCR Analysis)

From the protein data shown in Figure 2 and Figure 3, it is clear that Tff2 occurs in different molecular forms in the stomach and pancreas, respectively. In the stomach, a high-molecular-mass form of Tff2 is characteristic (Figure 2), whereas, in the pancreas, a low-molecular-mass form was observed (Figure 3). From previous data from the human stomach, it is well known that TFF2 binds strongly to the mucin MUC6 as a lectin via the evolutionary conserved terminal carbohydrate moiety GlcNAcα1→4Galβ (review: [34]).

**Figure 7:**
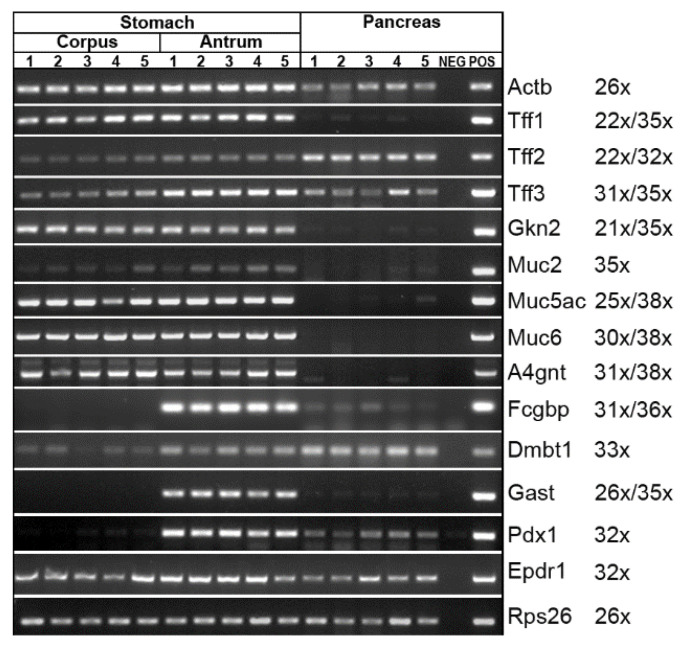
RT-PCR analysis. *Tff1*, *Tff2*, *Tff3*, *Gkn2*, *Muc2*, *Muc5ac*, *Muc6*, *A4gnt*, *Fcgbp*, *Dmbt1*, *Gast*, *Pdx1*, and *Epdr1* expression in the gastric corpus and antrum, respectively, as well as in the pancreas. The number of amplification cycles is given in parentheses (if different for stomach and pancreas). As controls, expression of *Actb* and *Rps26*, respectively, was monitored.

Here, using expression analysis, we investigate the possible reason for the different Tff2 forms in the stomach and pancreas, respectively (Figure 7). We even compared the two characteristic regions of the stomach, i.e., the corpus and the antrum. We hypothesize that possibly Muc6 and/or the GlcNAcα1→4Galβ carbohydrate moiety of Muc6 are missing in the pancreas so that lectin-mediated Tff2/Muc complexes cannot form. A comprehensive expression profiling was performed including transcripts encoding TFF peptides (*Tff1*, *Tff2*, *Tff3*) and polypeptides/proteins known to be associated with TFF peptides (*Gkn2*, *Muc6*, *Fcgbp*). Furthermore, expression of the mucins *Muc2* and *Muc5ac* was included, as well as the enzyme responsible for the synthesis of the terminal GlcNAcα1 sugar moiety in Muc6 (*A4gnt*). In addition, and as controls, expression of *Dmbt1* (a cysteine-rich glycoprotein with a role in mucosal innate immunity), the hormone gastrin (*Gast*) typical of the gastric antrum, the transcription factor *Pdx1* typical of the gastric antrum and pancreas, and the ependymin-related protein (*Epdr1*/UCC1; [64,65]) was monitored. The major results are (Figure 7): (i) *Tff2* (together with *Tff3*) is the typical TFF gene expressed in the murine pancreas. (ii) Neither *Muc6* nor *A4gnt* expression was detectable in the pancreas within this study. (iii) In contrast, *Muc6* and *A4gnt* transcripts were easily detected in the stomach. (iv) *Fcgbp* and *Gast* are typically expressed in the gastric antrum.

**Figure 8:**
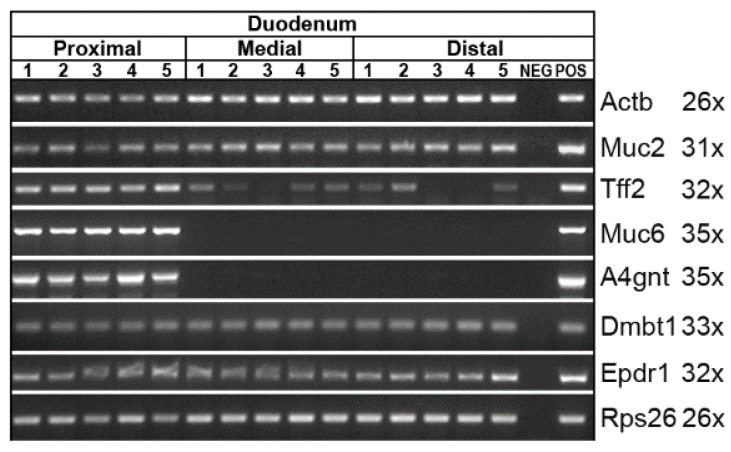
RT-PCR analysis. *Muc2*, *Tff2*, *Muc6*, *A4gnt*, *Dmbt1*, and *Epdr1* expression in the proximal, medial, and distal portions of the murine duodenum. The number of amplification cycles is given in parentheses. As controls, the expression of *Actb* and *Rps26*, respectively, was monitored.

In addition, the duodenum was analyzed by expression profiling concerning *Tff2*, *Muc2*, Muc6 and *A4gnt* (Figure 8). As controls, the expression of *Dmbt1* and *Epdr1* was monitored. As Brunner glands are restricted to the proximal duodenum [39], the duodenum was divided into the proximal, medial, and distal regions. There is a clear difference between the proximal and more distal parts concerning *Muc6* and *A4gnt*, i.e., *Muc6* and *A4gnt* transcripts were detectable in the proximal third only (Figure 8). In contrast, *Tff2* expression was detected in all parts, with a preponderance in the proximal part. All the other transcripts investigated showed an even distribution between the different regions.

### 2.3. Protein Analysis of the Murine Gastric Corpus, Gastric Antrum, and Pancreas

**Figure 9:**
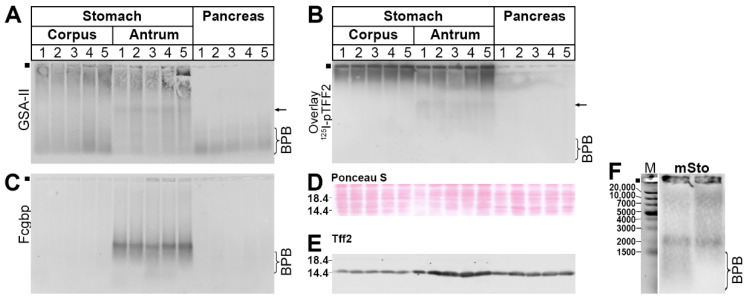
Western blot analysis and in vitro binding study. 1% AgGE and Western blot analysis of TRIzol extracts from 5 different samples (30 µg protein per lane) of the murine gastric corpus, gastric antrum, and pancreas, respectively (**A**–**C**). (**A**) Analysis concerning Muc6 (lectin GSA-II); arrow: antrum-specific signals. (**B**) Overlay assay with ^125^I-pTFF2; arrow: antrum-specific signals. (**C**) Analysis concerning Fcgbp. (**D**,**E**) Loading controls of the same samples after reducing SDS-PAGE: protein staining with Ponceau S (**D**) and Western blot concerning Tff2 (**E**), respectively. (**F**) Controls: Relative standard: DNA ladder (base pairs), murine gastric extracts (before SEC and fraction B9 from Figure 2; analysis concerning Fcgbp). The start (dot) and the dye bromophenol blue (BPB) are marked.

The RT-PCR analysis (Figure 7) revealed that the stomach and pancreas differ in the expression of *Muc6* and *A4gnt,* and *Fcgbp* is restricted to the gastric antrum. These results were checked for the protein level by Western blot analyses of TRIzol extracts (Figure 9).

Clearly, the GSA-II detected high-molecular-mass products in the stomach only. Of note, there is also a signal, which appeared in the gastric antrum only (see arrow in Figure 9A). We also performed an in vitro binding study of these extracts with ^125^I-pTFF2 (overlay assay; Figure 9B). Here, the ^125^I-pTFF2 bound predominantly to an ultrahigh-molecular-mass region (gel pocket) but also faintly to a region exclusively in the gastric antrum (see arrow in Figure 9B). Fcgbp is restricted to the gastric antrum (Figure 9C). The loading control is shown in Figure 9D. The relative Tff2 content of the samples was analyzed in Figure 9E. Remarkably, the relative Tff2 content in the pancreas is higher than in the gastric corpus. The highest Tff2 concentrations are found in the gastric antrum.

## 3. Discussion

### 3.1. The Predominant Gastric Tff2 Form Is Different When Compared with the Pancreatic Form

From a comparison of the elution profiles after the SEC, it is clear that the predominant Tff2 form in the murine stomach (Figure 2A) is different from that in the pancreas (Figure 3A). Gastric Tff2 mainly occurs in a high-molecular-mass form (Figure 2A), whereas pancreatic Tff2 is detectable in a low-molecular-mass form only (Figure 3A). Generally, this situation is comparable to that in the porcine stomach and pancreas, respectively [61].

Based on results from human [43], porcine [62] and *X. laevis* stomach extracts [36], the high-molecular-mass form of murine gastric Tff2 is expected to represent a non-covalent complex between Tff2 and the mucin Muc6, due to a lectin interaction. In the latter, the conserved O-linked terminal αGlcNAc carbohydrate moiety plays an essential role (review: [34]). In agreement with this, we also show the binding of radioactively labeled porcine TFF2 with gastric Muc6 (overlay assay), which was denatured and depleted from the endogenous Tff2 by preceding boiling with β-mercaptoethanol (Figure 6B). The positive binding after reduction is also a strong indication that TFF2 binds via the sugar moiety, as typical of a lectin.

Furthermore, murine gastric Tff2 can be released from the Tff2/Muc6 complex by boiling in SDS (Figure 2B). Of note, the release of murine Tff2 by boiling in SDS is not complete, and significant amounts (about 30%) of Tff2 are still bound to Muc6, as they appear in the high-molecular-mass region and even the gel pocket (Figure 2C). Only after denaturing extraction with TRIzol Reagent, the Tff2 could be released completely from the Tff2/Muc6 complex [66]. This is reminiscent of porcine stomach extracts, where the majority of TFF2 could not be separated from MUC6 by boiling in SDS [61,62]. In contrast, in human stomach extracts, TFF2 was completely released by boiling in SDS [43]. Currently, the reason why TFF2 is bound much stronger to MUC6 in the murine and porcine gastric mucus when compared with human mucus is not clear. In principle, the N-glycosylation of human TFF2 could be the reason, as neither murine nor porcine Tff2/TFF2 are glycosylated. Furthermore, in porcine TFF2, the formation of a non-covalently linked homodimer [61] might be an additional factor responsible for the extreme stability of the porcine TFF2/MUC6 complex against boiling in SDS, and even denaturing extraction with TRIzol [62]. The biological role of the unusual stability of the murine, and particularly the porcine, TFF2/MUC6 complex has not been clarified, but it is tempting to speculate that this is an evolutionary adaptation of the gastric mucus barrier to the different nutritional habits of these animals when compared with humans.

The low-molecular-mass form of Tff2 observed in the pancreas (Figure 3A) probably represents a monomer (Figure 3B). Of note, after the non-reducing SDS-PAGE, Tff2 appears as a triple band (major: 18k; minor: 16k, 14k; Figure 3B). The 18k band probably represents a circular form, which is formed by a disulfide bridge between Cys-6 and Cys-104 (Figure 1) [66]. This disulfide bridge has been reported to be particularly sensitive against reduction [67], which might explain the occurrence of the minor 16k and 14k bands, the latter being similar to the monomeric band (Figure 3B). In all three bands, Tff2 was identified by proteome analysis (bands 2–4 in Figure 4D). Furthermore, Tff2 was also identified after reducing SDS-PAGE (band 1 in Figure 4D). Remarkably, here the peptide LVEGEKPSPCR was identified. Based on the known cDNA sequence, this is expected to represent the N-terminal of the Tff2. To our knowledge, this is the first time that the mature N-terminal of murine Tff2 has been determined, indicating cleavage of the precursor after Ala-19. This is somewhat surprising as, by comparison with porcine TFF2, one might have expected the N-terminal to be EKPSPCR—assuming cleavage of the precursor after Gly-23. However, the cleavage after Ala-19 would be perfectly in agreement with the classical “(-3,-1) rule” according to von Heijne [68].

As a next step, we performed an expression analysis (RT-PCR) of gastric and pancreatic specimens (Figure 7) in order to clarify why, in the pancreas, the high-molecular-mass Tff2/Muc6 complex is lacking (see Figure 3). In contrast to the stomach, both *Muc6* and *A4gnt* transcripts were not detectable in the pancreas in this study (Figure 7). Thus, the high-molecular-mass complex cannot form in the pancreas anymore, and Tff2 exists only in the low-molecular-mass form.

The RT-PCR data (Figure 7) were checked, in part, on the protein level (Figure 9). In agreement with the results from the RT-PCR analysis, high-molecular-mass reactivity for GSA-II (recognizing also Muc6) is restricted to the stomach (Figure 9A). Furthermore, ^125^I-labeled TFF2 is predominantly bound to the ultrahigh-molecular-mass region of the stomach, which is typical of binding to Muc6. Furthermore, Fcgbp could be detected in the gastric antrum only, which again is in agreement with the RT-PCR analysis (Figure 7), as well as with previous reports in mice and humans [66,69]. This is a strong indication that the gastric mucus layer is not uniform and differs in the corpus when compared with the antrum. In particular, Fcgbp is considered a component of the first-line innate immune defense, which seems to be of special importance to the gastric antrum [66].

### 3.2. Tff2 Forms in the Duodenum

Generally, the situation in the duodenum is comparable to that in the stomach. In the extracts from the total duodena, we identified predominantly the high-molecular-mass form and only relatively little monomeric Tff2 (Figure 5A).

The high-molecular-mass form of Tff2, representing a Tff2/Muc6 complex, can easily form in the proximal duodenum, as here, Tff2, as well as Muc6 and A4gnt, are expressed (Figure 8). Tff2 can be released from the Tff2/Muc6 complex by boiling in SDS (Figure 5C), indicating a non-covalent interaction. In agreement with this, radioactively labeled Tff2 binds to total duodenal Muc6 (overlay assay; Figure 5E), indicating again the formation of a non-covalent Tff2/Muc6 complex. Expression of *Tff2*, *Muc6*, and *A4gnt* is known to occur in the Brunner glands, which are localized in the proximal duodenum only and are usually not found beyond the entrance of the pancreatic duct, i.e., the ampulla of Vater [39,70]. The results from the RT-PCR analysis (Figure 8) are in agreement with this. However, the distance Brunner glands extend distally is highly variable between species [70].

Of special note, the medial and the distal parts of the duodenum are devoid of both *Muc6* and *A4gnt* transcripts, but there is a somewhat weaker, but significant, *Tff2* expression (Figure 8). This might explain why total duodenal extracts also contain little monomeric Tff2 (Figure 5A,B). In addition, monomeric Tff2 could also reach the duodenum from the pancreas via the pancreatic duct and the ampulla of Vater, the latter being a prominent location for adenoma and carcinoma in humans [71].

### 3.3. Possible Receptor-Mediated Protective Function of Pancreatic Tff2 in the Mouse

Clearly, pancreatic Tff2 is not associated with Muc6 (Figure 3). Only in the ampulla of Vater, which forms the entrance of the juncture of the common bile duct and the main pancreatic duct into the duodenum, pancreatic Tff2 might interact with Muc6, similarly as in humans. In the latter, MUC6, but not TFF2, is synthesized in this organ [7,71]. Thus, the question arises on the function of monomeric Tff2, particularly in the exocrine pancreas, but also the medial and distal duodenum.

**Figure 10:**
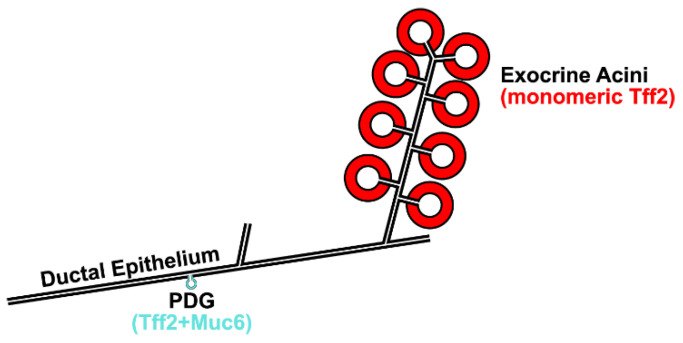
Schematic structure of the exocrine pancreas (not drawn to scale). Ductules from a group of acini converge to an intralobular duct, which is connected to interlobular and collecting ducts. Shown is the predominant synthesis of Tff2 in the acini (red), and its trace amount co-expression, together with Muc6, in the pancreatic duct glands (PDGs, turquoise). Of note, the total mass of PDGs is much smaller when compared with that of the exocrine acini.

The delicate pancreatic ductal epithelium is in contact with a cocktail of food-digesting enzymes, and it also produces essential constituents of the pancreatic juice, e.g., HCO_3_^−^ ions (maintaining a pH of about 8), as well as water. Thus, maintaining the integrity of this epithelium is extremely important. Monomeric Tff2 from the acini would be perfectly suited to protect the murine pancreatic ductal system (Figure 10) after damage, in particular after food ingestion, by promoting its rapid repair by restitution. Furthermore, monomeric Tff2 could protect the ductal epithelium, also, as an anti-inflammatory factor [23] by preventing inflammation, the latter being a known pre-requisite for carcinogenesis.

The pancreatic ductal epithelium, but not the terminal ducts, undergoes continuous self-renewal from stem and precursor cells located in the PDGs (Figure 10) [21]. Here, only trace amounts of Tff2 and Muc6 are secreted (Figure 10) [21,72], which might protect the content of the PDGs by a mucus plug (probably the formation of a Tff2/Muc6 complex). However, such trace amounts of a presumable Tff2/Muc6 complex were not detected with the methods used (Figure 3). Of special note, the migration of cells from the PDGs to populate the ductal epithelium is dependent on Tff2 [21].

*Tff2*^KO^ mice with a mutated KRAS^G12D^ background develop pancreatic mucinous neoplasm, which resembles human intraductal papillary mucinous neoplasm (IPMN), the latter being one of the precursor lesions of pancreatic ductal adenocarcinoma [60]. These data strongly suggest a protective/reparative function of monomeric Tff2 for the murine pancreatic ductal epithelium, e.g., by promoting restitution and inhibiting inflammation. Both effects would require receptors (potential candidates: [4,25,73,74]). In addition, monomeric Tff2 could also play a role during pancreatic organogenesis and lipid metabolism.

Remarkably, the situation in humans might be somewhat different. Here, only minute amounts of TFF2 were detectable [19,20,75], but there are multiple reports of significant pancreatic MUC6 expression. Thus, the TFF2/MUC6 ratio seems to be very different in humans and mice, i.e., it is probably much higher in the mouse. This might be indicative of different protection mechanisms in humans (mucous protection) and mice (monomeric Tff2-triggered protection).

In the future, it will be an ambitious aim to unambiguously identify the various TFF2 receptors, including their carbohydrate moieties. Of note, the latter probably differ in various cell types, as glycosylation patterns are dynamic and also cell type-specific [63]. The characterization of glycosylated TFF2 receptors might open, also, new therapeutic options as well as allow the growth of functional pancreas-derived 3D organoids [76].

## 4. Materials and Methods

### 4.1. Animals

The animal care and experimental procedures were conducted in compliance with Directive 2010/63/EU of the European Parliament and the Council of 22 September 2010 on the protection of animals used for scientific purposes, the German Animal Welfare Act, and the regulations on the welfare of animals used for experiments or for other scientific purposes (in their currently valid versions). In the course of these studies, adult wild type animals with a mixed 129/Sv and C57BL/6 background were used as described, i.e., they were fed ad libitum and non-fasted [77].

### 4.2. Extraction of Proteins, Protein Purification by SEC

Total stomachs, pancreata, and duodena, respectively, from 3–9 animals were collected and extracted with a 5- to 15-fold amount (*w*/*v*) (5-fold: stomach; 6-fold: duodenum; 15-fold: pancreas) of buffer (30 mM of NaCl, 20 mM of Tris-HCl pH 7.0 plus protease inhibitors) in a Precellys 24 lyser/homogenizer, similarly as described previously in detail (aqueous extracts) [29,78]. Furthermore, the proteins were extracted using TRIzol Reagent (Ambion by Life Technologies, Carlsbad, CA, USA) as described previously (TRIzol extracts) [66].

The aqueous extracts (5 mL each) were fractionated by SEC with the ÄKTA FPLC system (Amersham Biosciences, Freiburg, Germany) as described (fraction numbering: A1-A12, B1-B12, etc.) using the following columns [61]: HiLoad 16/600 Superdex 75 prep grade (S75HL; 20 mM of Tris-HCl pH 7.0, 30 mM of NaCl plus protease inhibitors; flow rate: 1.0 mL/min; 2.0 mL fractions) or HiPrep 16/60 Sephacryl S-500 High Resolution (S-500; 20 mM of Tris-HCl pH 7.0, 30 mM of NaCl plus protease inhibitors; flow rate: 0.5 mL/min, 2.0 mL fractions).

### 4.3. SDS-PAGE, AgGE, and Western Blot Analysis

Denaturing SDS-PAGE under reducing and non-reducing conditions, respectively, native AgGE, and Western blot analysis were described in detail previously [32,66,78,79]. The transfer of proteins to the nitrocellulose membrane was checked by staining with 0.2% Ponceau S (Carl Roth GmbH + Co., KG, Karlsruhe, Germany) in 3% trichloroacetic acid. The gels, after the non-reducing SDS-PAGE, were subjected to post-in-gel reduction with 1% mercaptoethanol at 50 °C for 5 min, according to a previous report [61]. As a relative standard for non-denaturing AgGE, the GeneRuler 1kb Plus DNA Ladder (Thermo Fisher Scientific Baltics UAB, Vilnius, Lithuania) was used as described previously [80].

The murine Tff1 and Tff2 were detected with the affinity-purified polyclonal antisera anti-mTff1-1 [81], anti-TFF2 (PA5-75670; Invitrogen by Thermo Fisher Scientific Baltics UAB, Vilnius, Lithuania), and anti-hTFF2-2 [62] (Figure 9E), respectively. The mucins Muc6 and Muc5ac were detected with the biotinylated lectins GSA-II from *G. simplicifolia* or WFA from *Wisteria floribunda*, respectively, as reported [42,61,63]. The Fcgbp was detected with a polyclonal antiserum against a fragment of rat Fcgbp kindly provided by Prof. Jürgen Seitz (Philipps University, Marburg, Germany) [82].

### 4.4. Identification of Proteins by Bottom-Up Proteomics

For the protein identification, 150 or 200 µL of fraction D6 (Figure 3) was concentrated, separated by non-reducing or reducing SDS-PAGE, and stained with Bio-Safe Coomassie Stain G-250, as described [62]; the gel bands were excised and subjected to tryptic digestion, followed by liquid chromatography coupled to electrospray ionization and tandem mass spectrometry (LC-ESI-MS/MS). The data obtained were processed and analyzed with a search engine as described in detail elsewhere [80].

### 4.5. Tff2 Binding Studies

The labeling of the porcine pancreatic TFF2 (pTFF2) with ^125^I was as described previously [62]. The pTFF2 was kindly provided by Dr. L. Thim (Novo Nordisk A/S, Maaloev, Denmark) [1]. Overlay assays with 125I-labeled pTFF2 were performed as described previously [62].

### 4.6. RNA Extraction, PCR Analysis

The isolation and purification of total gastric and duodenal RNA, respectively, (TRIzol Reagent; Ambion by Life Technologies, Carlsbad, CA, USA), pancreatic RNA (RNA Mini Kit, Bioline, Heidelberg, Germany), as well as RT-PCR (reverse transcriptase: Takara Bio Europe, Saint Germain en Laye, France), were as described in detail previously [81,83,84].

The specific primer pairs used for the RT-PCR have been published previously (*Actb*, MB2658/MB2659; *Epdr1* MB1890/1891; *Gast* MB2450/MB2451; *Muc2* MB2660/MB2661; *Pdx1* MB2464/MB2465; *Tff1* MD7/MD8; and *Tff2* MB2306/2307) [66,83,84,85] and are listed in Table 1 (*A4gnt*, *Dmbt1*, *Fcgbp*, *Gkn2*, *Muc5ac*, *Muc6*, *Rps26*, and *Tff3*). All the primer pairs used are intron spanning.

## Figures and Tables

**Table 1:** Oligonucleotides used for RT-PCR analysis and calculated size of the products.

**Genes** **Accession No.**	**Primer** **No.**	**Primer Pairs**	**Nucleotide** **Positions**	**Annealing T** **Size (bp)**
*A4gnt* NM_001077424.2	MB2430 MB2431	GAAGATTAGGCAGTGAGTTACCA TTAAGACGACACCACACCCG	2–24 897–878	60 °C 896
*Dmbt1* NM_001347632.2	MB2869 MB2870	GAACCGGCACAATGGGGATCT ATAGGACACTTCATCTGTGGGAAC	6–26 115–92	60 °C 110
*Gkn2* NM_025467.1	MB2732 MB2733	TTCTGGTGGTGCTGTCCATC TAGGCGACCCAAACAGGAAC	51–70 446–427	60 °C 396
*Fcgbp* NM_001122603.1	MB2448 MB2449	ATTCTGTGTCGCTGGTTCGT CAGTTGGCCATCCCAGTCAT	384–403 556–537	60 °C 173
*Muc5ac* NM_010844.3	MB2318 MB2319	TACCATGAACACCGCTCTGA GTTGGAGAGGAACTCGTTGG	146–165 718–699	58 °C 573
*Muc6* NM_001330001.2	MB2320 MB2321	CCCTCATGGCTGTGTATGAC TTGTGGTTCAAGTAGGTGCC	1389–1408 2223–2204	58 °C 835
*Rps26* NM_013765.2	MB1494 MB1495	CCAAAACCTGGAGATGAGGA CAGGCTACGGCAGAGATAGG	120–139 382–363	57 °C 263
*Tff3* NM_011575.2	MB2470 MB2471	GCTACCCCTCTGTCACATCG ATCAGCCTTGTGTTGGCTG	166–185 440–421	60 °C 275

## Abbreviations

AgGE	Agarose gel electrophoresis
FCGBP	IgG Fc binding protein
Gkn	Gastrokine
IL	Interleukin
PDG	Pancreatic duct gland
SEC	Size exclusion chromatography
SDS-PAGE	Sodium dodecyl sulfate-polyacrylamide gel electrophoresis
TFF	Trefoil factor family
